# A versatile *cis*-prenyltransferase from *Methanosarcina mazei* catalyzes both *C*- and *O*-prenylations

**DOI:** 10.1016/j.jbc.2021.100679

**Published:** 2021-04-17

**Authors:** Miyako Okada, Hideaki Unno, Koh-Ichi Emi, Mayuko Matsumoto, Hisashi Hemmi

**Affiliations:** 1 Graduate School of Bioagricultural Sciences, Nagoya University, Nagoya, Aichi, Japan; 2 Graduate School of Engineering, Nagasaki University, Nagasaki, Nagasaki, Japan; 3 Organization for Marine Science and Technology, Nagasaki University, Nagasaki, Nagasaki, Japan

**Keywords:** *cis*-prenyltransferase, *Methanosarcina mazei*, methanogen, archaea, crystal structure, *O*-prenylation, isoprenoid, cPT, *cis*-prenyltransferase, DMAPP, dimethylallyl pyrophosphate, FG, farnesyl-*sn*-glycerol, FPP, farnesyl pyrophosphate, IPP, isopentenyl pyrophosphate, PPi, inorganic pyrophosphate, TLC, thin-layer chromatography

## Abstract

Polyprenyl groups, products of isoprenoid metabolism, are utilized in peptidoglycan biosynthesis, protein *N*-glycosylation, and other processes. These groups are formed by *cis*-prenyltransferases, which use allylic prenyl pyrophosphates as prenyl-donors to catalyze the *C*-prenylation of the general acceptor substrate, isopentenyl pyrophosphate. Repetition of this reaction forms (*Z,E*-mixed)-polyprenyl pyrophosphates, which are converted later into glycosyl carrier lipids, such as undecaprenyl phosphate and dolichyl phosphate. MM_0014 from the methanogenic archaeon *Methanosarcina mazei* is known as a versatile *cis*-prenyltransferase that accepts both isopentenyl pyrophosphate and dimethylallyl pyrophosphate as acceptor substrates. To learn more about this enzyme’s catalytic activity, we determined the X-ray crystal structures of MM_0014 in the presence or absence of these substrates. Surprisingly, one structure revealed a complex with *O*-prenylglycerol, suggesting that the enzyme catalyzed the prenylation of glycerol contained in the crystallization buffer. Further analyses confirmed that the enzyme could catalyze the *O*-prenylation of small alcohols, such as 2-propanol, expanding our understanding of the catalytic ability of *cis*-prenyltransferases.

*Cis*-prenyltransferase (cPT) generally catalyzes the consecutive head-to-tail condensation of isoprene units and produces (*Z,E*-mixed)-polyprenyl pyrophosphates (Fig. 1*A*) (1, 2). The reaction starts with the transfer of a prenyl group from the first donor substrate, (all-*E*)-prenyl pyrophosphate such as farnesyl pyrophosphate (FPP), to the C4 carbon of the acceptor substrate isopentenyl pyrophosphate (IPP). The pyrophosphate group of the donor substrate is eliminated as inorganic pyrophosphate (PPi), concertedly with the C-C bond formation. This *C*-prenylation reaction yields prenyl pyrophosphate that is elongated by one isoprene unit with a *Z*-double bond, from which a second prenyltransfer to another IPP molecule proceeds. By repeating a similar condensation reaction, cPTs from bacteria and archaea typically produce a C_55_ product (undecaprenyl pyrophosphate), while eukaryotic cPTs usually yield longer (>C_70_) products. The products of cPTs are metabolized into glycosyl carrier lipids such as undecaprenyl phosphate and dolichyl phosphate, which are typically utilized for peptidoglycan biosynthesis and protein *N*-glycosylation, respectively. In addition, there are cPTs that give much longer or shorter products. Natural rubber is biosynthesized by the action of cPT from *Hevea brasiliensis* (3). Shorter (all-*Z*)-prenyl pyrophosphates such as neryl pyrophosphate, (*Z,Z*)-FPP, and nerylneryl pyrophosphate are produced from one dimethylallyl pyrophosphate (DMAPP) and 1 to 3 IPP molecules *via* the actions of plant cPTs and are used as the precursors of terpenes (Fig. 1*B*) (4, 5, 6).

**Figure 1:**
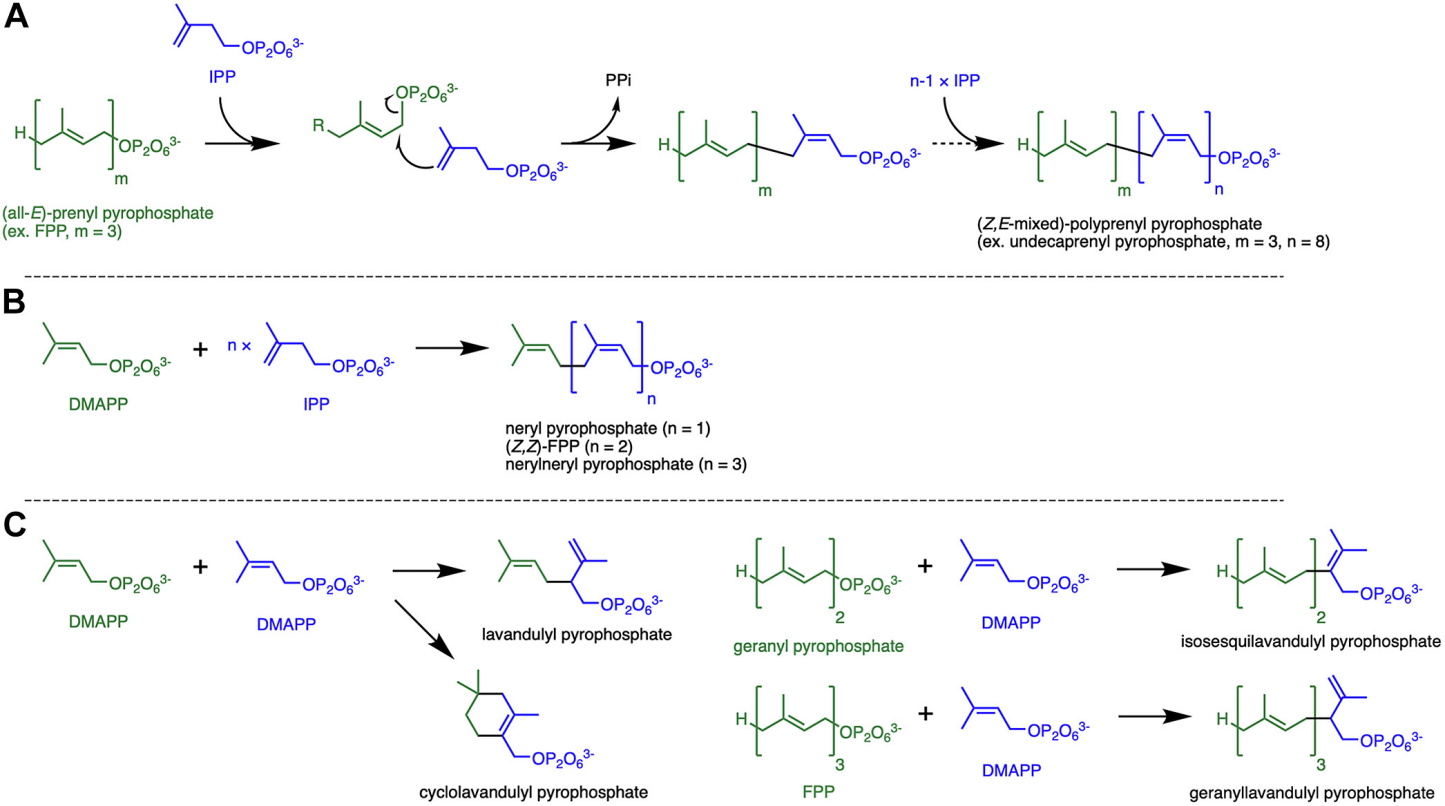
**Reaction schemes of cPTs.** Prenyl donor (for the initial reaction) and acceptor substrates are shown in *green* and *blue*, respectively. *A*, usual head-to-tail cPT reactions. *B*, head-to-tail cPT reactions that yield shorter (all-*Z*)-prenyl pyrophosphates. *C*, head-to-middle prenyl condensation reactions catalyzed by cPT homologs. cPT, cis-prenyltransferases.

Some cPT homologs, however, are known to accept prenyl pyrophosphates as an acceptor substrate and yield irregular products formed through head-to-middle prenyl condensation. For example, lavandulyl pyrophosphate synthase (7) and cyclolavandulyl pyrophosphate synthase (8) catalyze the condensation of two DMAPP molecules, and isosesquilavandulyl pyrophosphate synthase transfers a geranyl group from geranyl pyrophosphate to the C2 of DMAPP (Fig. 1*C*) (9). These irregular prenyltransferases have strict substrate specificities, and the mechanism of the catalysis for irregular *C*-prenylation has been elucidated in part by structural studies on the enzymes (10, 11, 12). On the other hand, some cPTs are known to catalyze both head-to-tail and head-to-middle prenyl condensation reactions. MA1831 and MM_0014, which are orthologous cPTs from the methanogenic archaea *Methanosarcina acetivorans* and *Methanosarcina mazei*, respectively, unusually accept both IPP and DMAPP as prenyl acceptor substrates (13, 14). The regular condensation reaction between FPP and IPP gives relatively short polyprenyl pyrophosphates, while the irregular reaction between FPP and DMAPP yields geranyllavandulyl pyrophosphate (Fig. 1*C*). Similar versatility in acceptor substrate recognition has also been reported for (*Z,Z*)-FPP synthase from *Solanum habrochaites* (15). Although the crystal structures of the plant enzyme are known, the reason for such a “loose” substrate specificity remains obscure.

In the present study, we solved the crystal structures of MM_0014. To understand the binding modes of different substrates, the enzyme crystals in the substrate-free and FPP-complex forms were soaked with either IPP or DMAPP, respectively. Some of the solved structures provided insight into the substrate preference for the versatile cPT. Interestingly, at least one structure revealed that the enzyme can catalyze an *O*-prenyltransfer reaction by the use of glycerol in the crystallization buffer as a prenyl acceptor substrate. Additional *in vitro* assays showed that the enzyme prenylates small alcohols, such as ethanol, 1-propanol, and 2-propanol, and also a water molecule as prenyl acceptors. These findings imply the role of the unique C-terminal structure of this enzyme on its specific substrate-binding mechanism.

## Results

### Structure of substrate-free MM_0014

**Figure 2:**
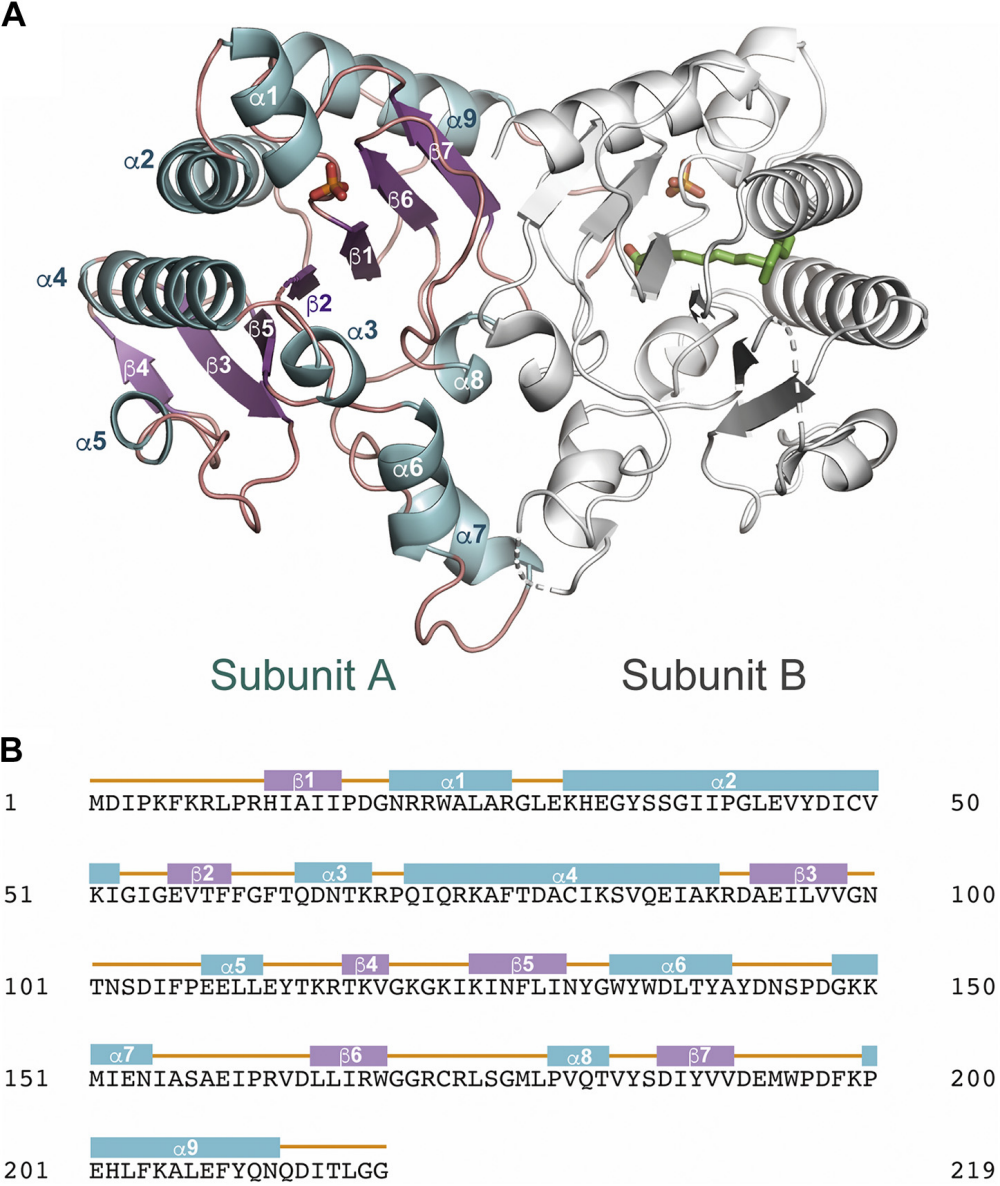
**Crystal structure of the cPT from *M. mazei*, MM_0014.***A*, dimer structure of MM_0014 in a *ribbon* representation. The α-helices and β-strands in subunit A are *cyan* and *purple*, respectively. Subunit B is *light gray*. Disordered regions are shown as *dotted lines*. Bound phosphates and palmitic acid are shown as *stick models*. *B*, amino acid sequence of the cPT from *M. mazei*, MM_0014. The α-helices (*cyan boxes*) and β-strands (*purple boxes*) are indicated.

**Figure 3:**
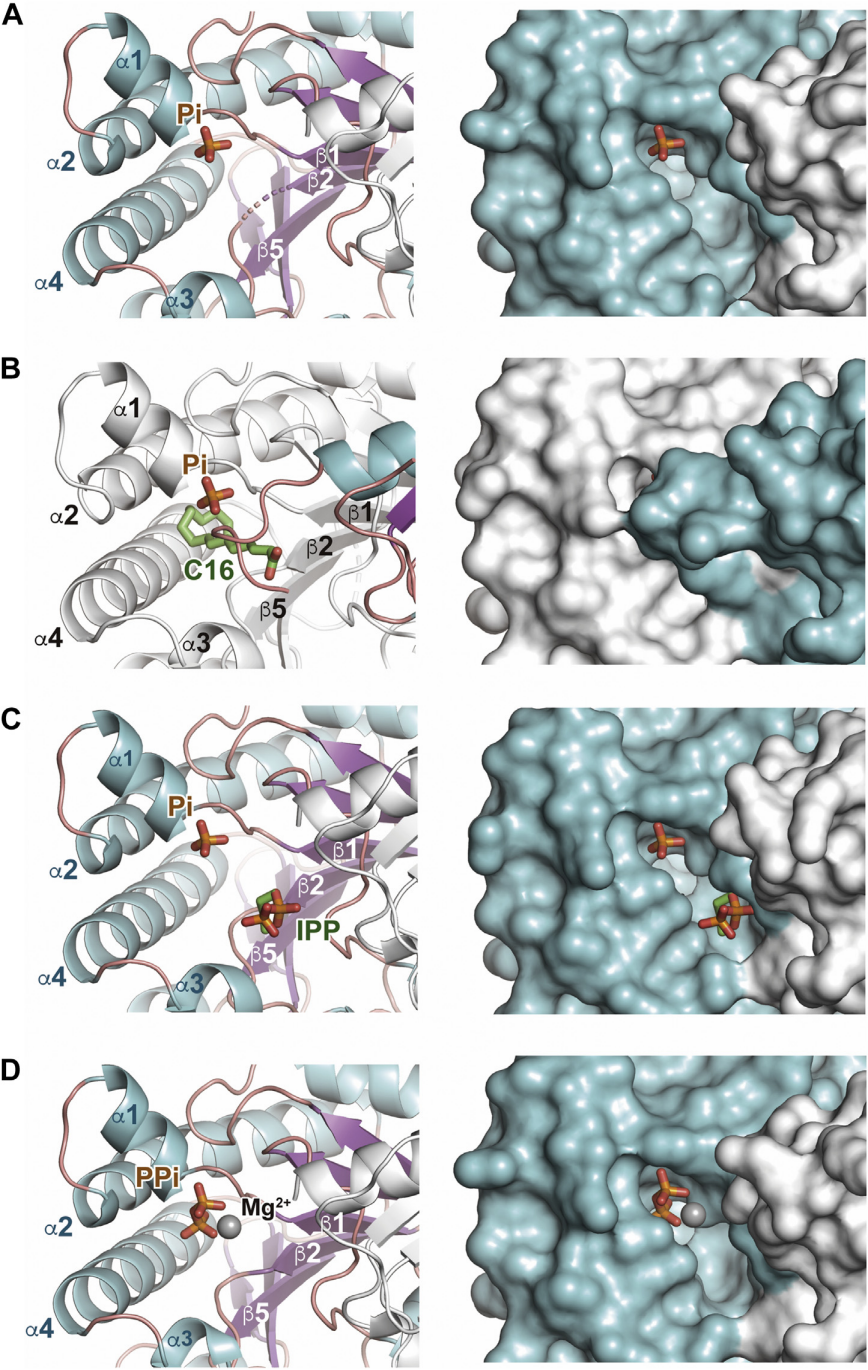
**Comparison of the ligand-binding cavities in complex structures of MM_0014.***Left* and *right panels* show the *ribbon model* and surface representations of the cavity structures, respectively. Subunits A and B of the surface representations are *cyan* and *light gray*, respectively. Ligand molecules (*stick models*) and Mg^2+^ ion (*sphere*) are shown. *A*, subunit A of the substrate-free structure. *B*, subunit B of the substrate-free structure. *C*, subunit A of the IPP-binding (free+IPP) structure. *D*, subunit A of the pyrophosphate-binding (free+PPi) structure.

Recombinant MM_0014 fused with an N-terminal thioredoxin-polyhistidine tag was expressed in *Escherichia coli* cells and purified *via* affinity chromatography. After cleavage of the tag sequence, which should have given the N-terminal sequence starting from GPGYQMDIPKFK where the methionine residue is the putative first residue of native MM_0014, the enzyme was further purified using gel-filtration chromatography and then crystallized in its substrate-free form. The asymmetric unit of the crystal with a C222_1_ space group contained two protein molecules that comprised a homodimer, which is considered to be the typical biological unit for general cPTs (1, 16) (Fig. 2*A*). Subunit A was disordered in the region upstream from Phe6, and subunit B was similarly disordered in this region, but also in the regions between Gly120 and Ile124, between Tyr142 and Lys149, and downstream from Gln213. In each subunit, a β-sheet was composed of parallel β7-β6-β1-β2-β5-β3 strands, and there was an antiparallel β4 strand that existed only in subunit A (Fig. 2*B*). The β-sheet was sandwiched by α-helices, and a narrow cavity was formed by α1, α2, α3, and α4 helices and β1, β2, and β5 strands. The overall structure of MM_0014 is in a ζ-fold (17), which is similar to those of so far reported cPTs (1, 16). An inorganic phosphate, which might have been bound throughout the purification process, existed at the opening of the cavity of each subunit (Fig. 3, *A* and *B*, and Fig. S1). Moreover, a long electron density patch was observed only in the cavity of subunit B (Fig. 3*B*, Figs. S1 and S2). Because the density was thought to be derived from a fatty acid bound to the recombinant enzyme during the expression in the host *E. coli* cells, palmitic acid was placed provisionally as the ligand because it fit the density well. Although the C-terminus of subunit B was disordered, the C-terminal structure was resolved in subunit A. The C-terminus of subunit A extended toward the cavity of subunit B and covered its cavity opening, where the carboxyl group of the terminal Gly219 residue interacted with a carboxyl oxygen of the purported palmitic acid ligand. In contrast, the cavity of subunit A was in an open conformation because the C-terminus of subunit B was deemed operationally disordered.

### Substrate binding of MM_0014

The crystals of substrate-free MM_0014 were soaked with either IPP or DMAPP to obtain its substrate-complex structures. When soaked with IPP overnight, an electron density consistent with an IPP molecule was observed in subunit A (Fig. 3*C*, Figs. S1 and S2), but not in subunit B where fatty acid was still bound. IPP was bound to the cavity opening of subunit A, which remained in an open conformation and bound an inorganic phosphate molecule. Superposition of the two subunits of the IPP-binding structure (designated as “free+IPP”) showed that a fatty acid ligand overlapped IPP and therefore might inhibit the binding of the substrate to subunit B (Fig. S3). It is also curious that no electron density derived from a Mg^2+^ ion was observed even though the crystallization buffer contained Mg^2+^. This might be because IPP was not precisely at the catalytically active position, as discussed later. In contrast, when soaked with DMAPP for 10 min, subunit A accommodated a pyrophosphate-Mg^2+^ complex (Fig. 3*D* and Fig. S1). Therefore, the crystal structure was designated as “free+PPi.” The binding site of the pyrophosphate molecule overlapped with that of the inorganic phosphate, not IPP, in the free+IPP structure. The electron density corresponding with the dimethylallyl moiety of DMAPP was sparse and barely visible, suggesting the possibility that MM_0014 rapidly hydrolyzed the allylic substrate with the aid of Mg^2+^, probably through Lewis-acid-assisted catalysis (Fig. S2). Based on these crystal structures, we presumed that, in MM_0014, the binding sites of the prenyl acceptor (IPP) and donors (allylic substrates) for the head-to-tail condensation (Fig. 1*A*) agree with those of previously studied cPTs (1, 16).

**Figure 4:**
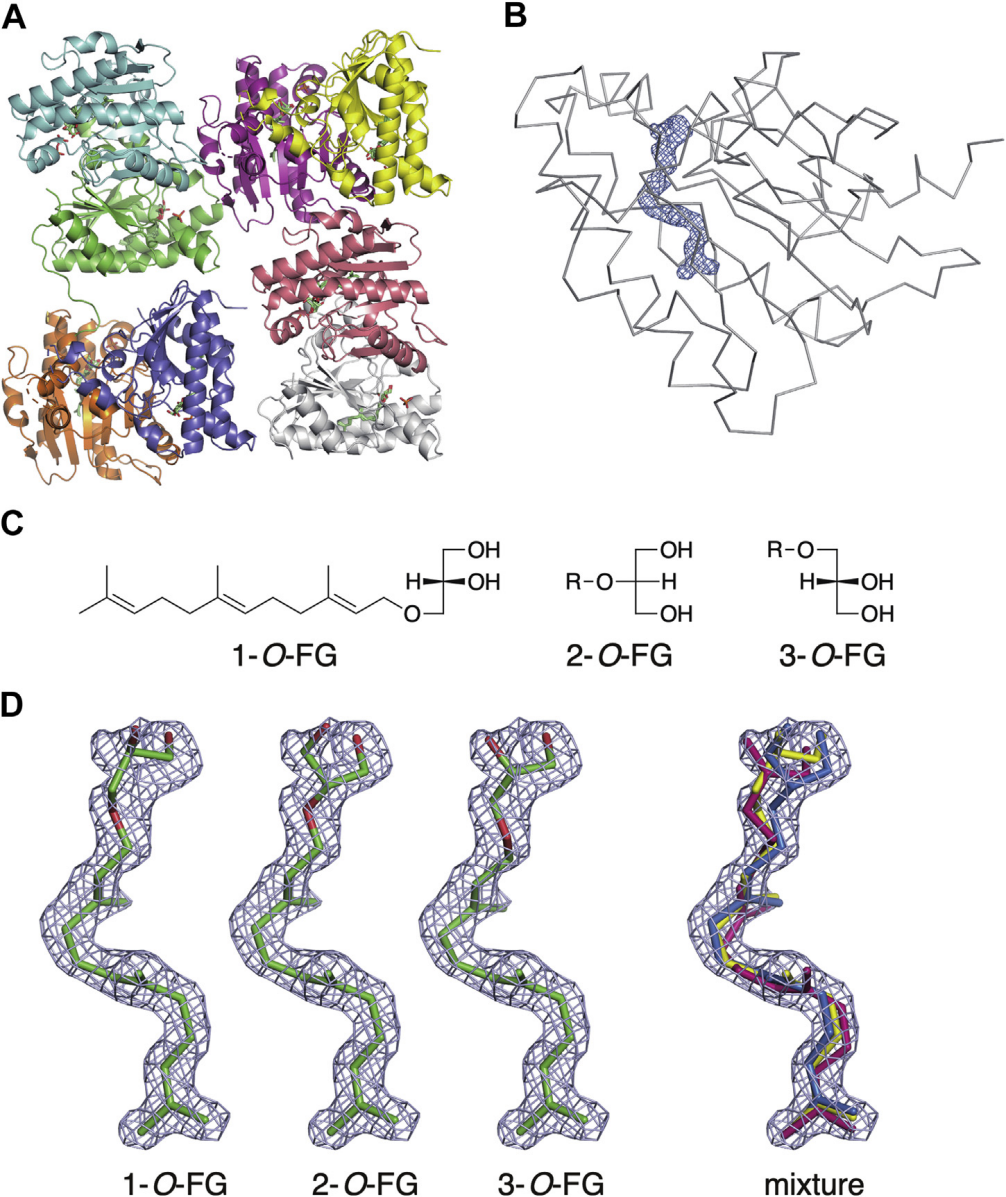
**FG-complex (co-FG) structure.***A*, four homodimer structures of the asymmetric unit are colored differently. FG and phosphate molecules are shown as *stick models*. *B*, *F*_o_-*F*_c_ omit electron map (*blue*) for FG bound to MM_0014. The contour level of the omit maps is 3σ. Main chain tracing representation of subunit B in the complex structure is colored in *light gray*. *C*, structures of FG isomers. R refers to a farnesyl group. *D*, superpositions of FG models with the *F*_o_-*F*_c_ omit electron map. The FG molecules fitted to the map are shown as *stick models* in *gray* mesh. The 1-*O*-FG, 2-*O*-FG, and 3-*O*-FG models, and an equal mixture of the models (1-*O*-FG, 2-*O*-FG, and 3-*O*-FG in *yellow*, *blue*, and *red*, respectively) are shown in order from *left* to *right*.

With a final aim of obtaining the ternary complex structures of MM_0014 with prenyl donor and acceptor substrates, a cocrystallization of MM_0014 with FPP and Mg^2+^ was performed using a different crystallization buffer. The asymmetric unit of the P2_1_2_1_2_1_ space group contains eight protein molecules that form four homodimers (Fig. 4*A*). Each protein molecule possessed an electron density that penetrated the cavity more deeply than the fatty acid ligand did in the substrate-free structure (Fig. 4*B* and Fig. S1). The shape of the electron density, however, did not fit well with FPP, particularly at the pyrophosphate group. It is also noteworthy that inorganic phosphate was still bound at the supposed prenyl-donor binding site and that no electron density was derived from Mg^2+^ ion. These facts suggest that the ligand was actually a derivative of FPP. However, the electron density was obviously longer than farnesol (FOH), which can be produced from the hydrolysis of FPP. Thus, we speculated that the ligand was derived from the prenyltransfer reaction between FPP and glycerol because the crystallization buffer contained glycerol as a cryoprotectant. We constructed models of the possible products from the reaction, *i.e.*, 1-*O*-farnesyl-*sn*-glycerol (FG), 2-*O*-FG, and 3-*O*-FG (Fig. 4*C*), and fitted them with the electron density (Fig. 4*D*). A 1:1:1 mixture of the models seemed to fit better than each model, suggesting that different *O*-prenylation products are formed and bound in MM_0014.

**Figure 5:**
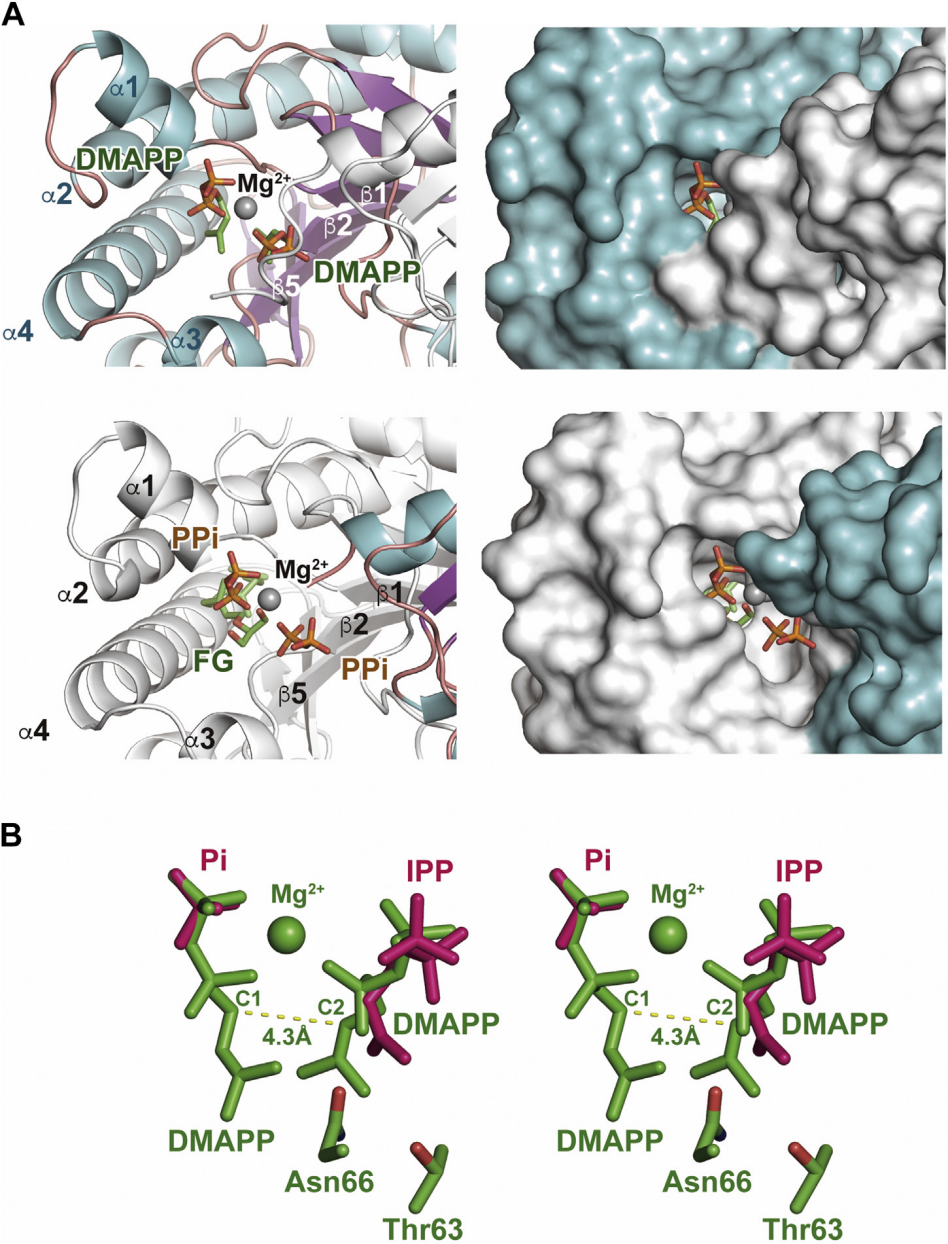
**FG-complex structure soaked with DMAPP (co-FG+DMAPP).***A*, ligand-binding cavities in an FG-binding subunit (only *sn*-2-*O*-FG is shown) and a DMAPP-binding subunit are shown in the *upper* and *lower panels*, respectively. *Left* and *right panels* show a *ribbon* model and surface representations of the cavity structures, respectively. Color representations of the panels are the same as those in Figure 3. *B*, stereo view of the super position of DMAPP-binding and IPP-binding cavities (co-FG+DMAPP and free+IPP, respectively). Structures of DMAPP- and IPP-complexes are colored *green* and *red*, respectively. DMAPP, IPP, phosphate molecules, and Thr63 and Asn66 residues are shown as stick models. Mg^2+^ ion is shown as a *sphere*.

The FG-complex crystals of MM_0014 (designated as “co-FG”) were soaked with DMAPP for 39 min to elucidate the binding mode of DMAPP at the prenyl acceptor-binding site, because the recognition of DMAPP as the prenyl acceptor substrate is required for the head-to-middle condensation. As a result, surprisingly, by replacing the FG ligand, two DMAPP molecules were found bound to four of the eight protein molecules in the asymmetric unit (Fig. 5*A*, Figs. S1 and S2). Therefore, the crystal structure was designated as “co-FG-DMAPP.” DMAPP molecules were accommodated in both the prenyl donor- and acceptor-binding sites, and a Mg^2+^ ion was coordinated between the pyrophosphate group of DMAPP at the donor-binding site and the α-phosphate of DMAPP at the acceptor-binding site (Fig. 5*A*, upper panels). These DMAPP-binding subunits were all in a closed conformation. In the other four protein molecules, FG and pyrophosphate were still bound in the donor-binding site, while a Mg^2+^ ion was coordinated between the pyrophosphate and α-phosphate of another pyrophosphate that was bound to the acceptor-binding site (Fig. 5*A*, lower panels). The DMAPP-binding protein molecule formed a dimer, either alone (one of four dimers in the asymmetric unit) or with an FG-binding protein molecule in an open conformation (two of four dimers). The remaining dimer consisted of two FG-binding protein molecules, while only one subunit held an inorganic phosphate at the acceptor-binding site in a closed conformation. The distance between the C1 of DMAPP at the donor-binding site and the C2 of DMAPP at the acceptor-binding site, which corresponded to the C-C bond-forming atoms in the head-to-middle condensation catalyzed by some cPT homologs, was 4.3 Å (Fig. 5*B*). This distance seems somewhat long, which could be why this structure could be solved without the complete processing of either a prenyltransfer or a hydrolysis reaction. The distance, however, could become somewhat shorter when the donor substrate is FPP, which yields geranyllavandulyl pyrophosphate from the reaction of MM_0014 with DMAPP. In addition, the fact that highly conserved asparagine and threonine residues (Asn66 and Thr63 in MM_0014), which are reported to be involved in proton elimination (18), exist in close proximity to a methyl group of DMAPP at the acceptor-binding site agrees with the supposed position of proton elimination in the formation of geranyllavandulyl pyrophosphate (14).

Comparison of the DMAPP-binding and IPP-binding cavity structures provided insight into the substrate preference of MM_0014. Superposition of the structures showed that the C4 of IPP, which is the atom involved in the C-C bond formation in the head-to-tail condensation, nearly overlaps the C2 of DMAPP (Fig. 5*B*). The atom positions, however, are inconsistent with the substrate preference of MM_0014, where IPP is much more reactive than DMAPP as the prenyl acceptor (13). In fact, the position of IPP seems unsuitable for the reaction. Because of the absence of a Mg^2+^ ion, the pyrophosphate group of IPP is misaligned by ∼3 Å from that of DMAPP. If an allylic substrate binds to the donor-binding site, IPP is considered to move deeper into the cavity because its pyrophosphate group would be coordinated to Mg^2+^ along with the allylic substrate. With such a suitable binding position, the C4 of IPP must move closer to C1 of the allylic substrate, and the C2 of IPP, from which a proton would be eliminated, must exist near the conserved residues Asn66 and Thr63, which are probably involved in proton elimination (18).

### *O*-prenylation catalyzed by MM_0014

**Figure 6:**
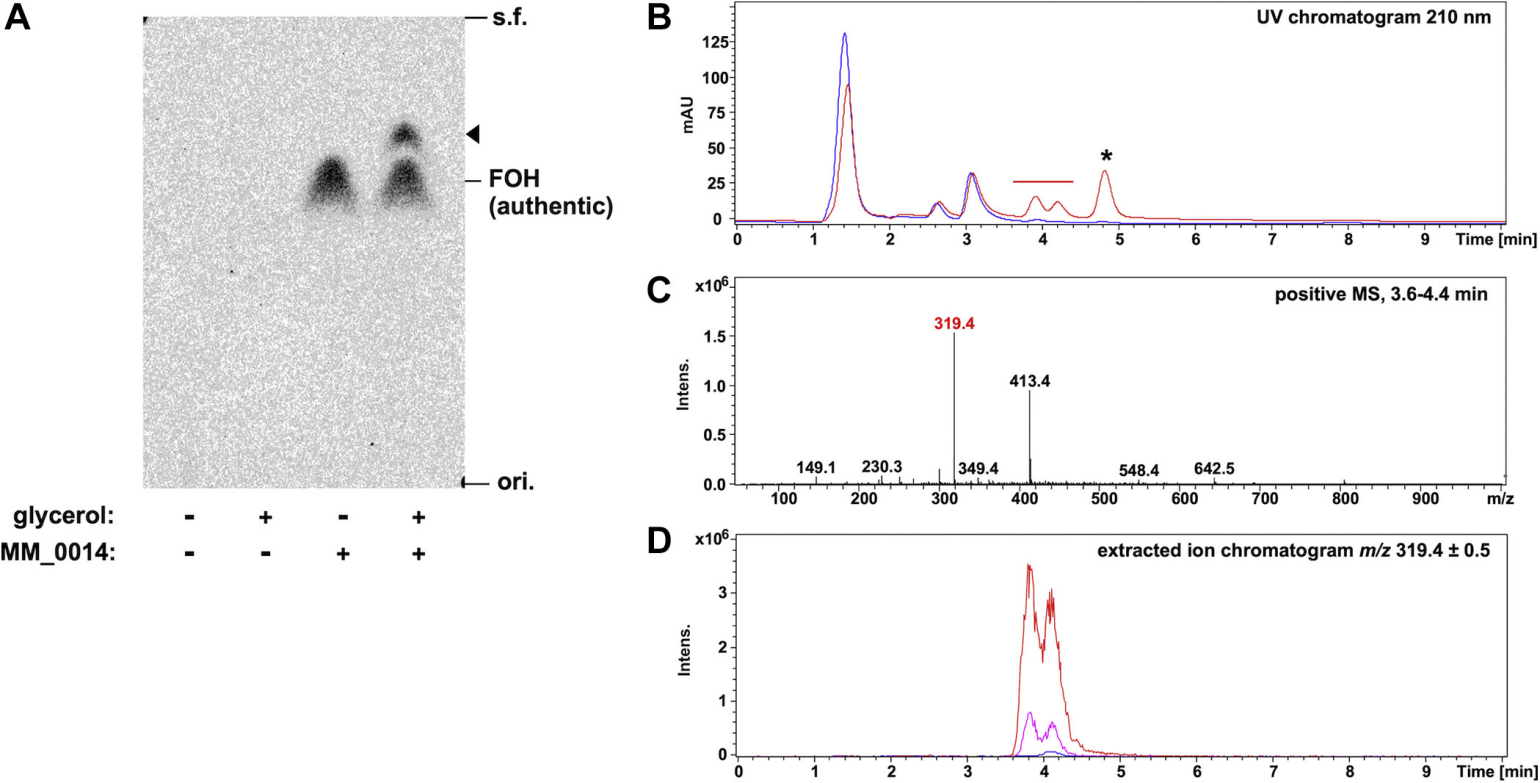
**Radio-TLC and LC-ESI-MS (positive-ion mode) analyses of MM_0014 reaction products from FPP and 25% (v/v) glycerol.***A*, TLC autoradiogram of pentane-extractable products from the reaction with [^14^C]FPP and glycerol. An *arrowhead* indicates the radioactive spot presumably derived from FG. *B*, UV chromatograms at 210 nm from LC-ESI-MS analyses. *Red* and *blue lines* represent the chromatograms from the reaction with or without MM_0014, respectively. Peaks observed only in the sample from reaction with MM_0014 were possibly derived from prenylated products, while that at ∼4.8 min (with an *asterisk*) was not coeluted with any specific positive ions. *C*, positive mass spectrum of eluent at 3.6 to 4.4 min (indicated with a *red bar* in *B*). The ion of *m/z* 319.4 corresponds well with [FG+Na]^+^, while that of *m/z* 413.4 was likely derived from a contaminated detergent and was detected throughout the chromatogram. *D*, extracted ion chromatograms of *m/z* 319.4 (in a range of ±0.5). *Red* and *blue lines* represent the sample from reaction with or without MM_0014, respectively, while the *pink line* represents a fivefold diluted sample with MM_0014.

To confirm if the *O*-prenyltransfer reaction is catalyzed by MM_0014, ^14^C-labeled FPP was reacted with an excess amount of the enzyme in the absence and presence of 25% (v/v) glycerol. After reaction, nonpolar products were extracted *n*-pentane and then analyzed by reversed-phase thin-layer chromatography (TLC) (Fig. 6*A*). Even in the absence of glycerol, a radiolabeled spot (*R*_f_ = 0.67) comigrated with authentic FOH was detected, suggesting that MM_0014 can catalyze hydrolyzation of FPP. Along with the spot, another radiolabeled spot (*R*_f_ = 0.76) was detected only in the sample from the enzyme reaction with glycerol, which suggested that the spot was derived from FG. In contrast, no pentane-extractable products were detected when the enzyme was removed from the reaction.

Next, nonlabeled FPP was reacted with the enzyme in the presence of 25% (v/v) glycerol, and the reaction products were analyzed *via* LC-ESI-MS following extraction with *n*-pentane (Fig. 6*B*). As a result, some UV peaks eluted at 3.6 to 5.0 min emerged only in the presence of MM_0014. The two UV peaks at 3.6 to 4.4 min were coeluted with a positive ion with *m/z* of 319, which correspond to FG-Na^+^ adduct ion (Fig. 6*C*), while any specific ions were not coeluted with the peak at ∼4.8 min. No ions obviously derived from FOH were detected under the conditions of the analysis. The separation of two peaks at 3.6 to 4.4 min was also observed in the extracted ion profile of *m/z* 319.4 ± 0.5 (Fig. 6*D*), which was unlikely the result of sample overloading because a diluted sample also gave two peaks. This suggested the production of the mixture of 1-*O*-FG, 2-*O*-FG, and 3-*O*-FG as expected from the structural study (Fig. 4*D*), because the enantiomeric 1-*O*-FG and 3-*O*-FG could not be separated under the conditions used for LC. The approximately 1:1 ratio of the peaks suggests that MM_0014 has a biased regio-specificity for *O*-prenylation, not having the same reactivity against all the hydroxy groups of glycerol.

**Figure 7:**
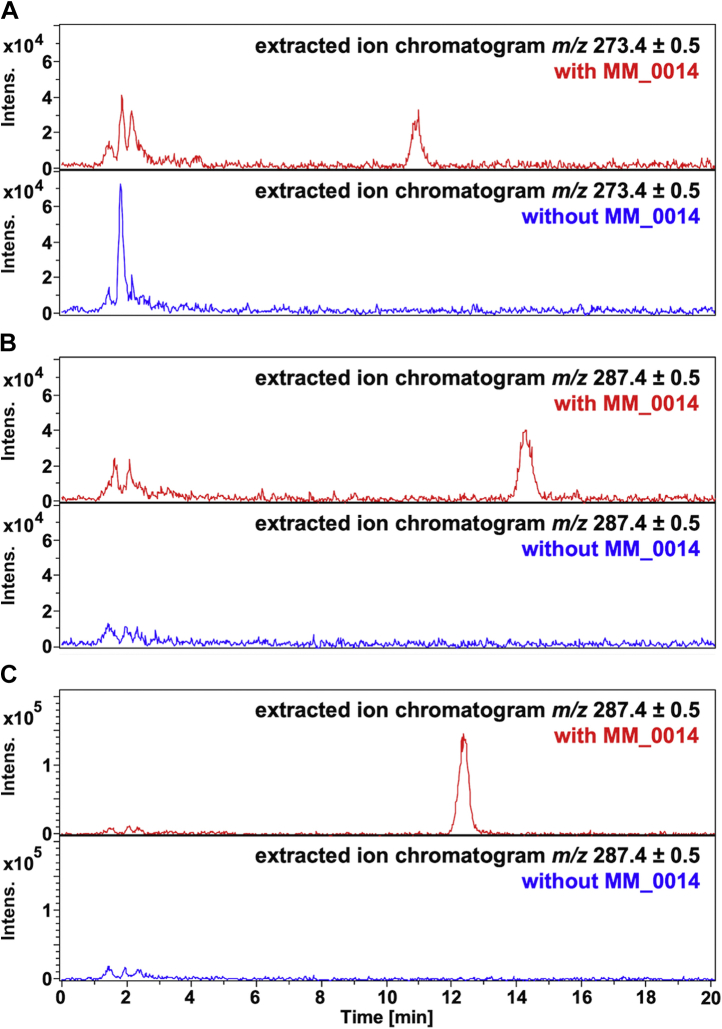
**LC-positive ESI-MS analysis of MM_0014 reaction products from FPP and 10% (v/v) small alcohols.** Shown are extracted ion chromatograms from the analyses of reaction products from FPP and ethanol (*A*), 1-propanol (*B*), or 2-propanol (*C*). *Upper* (with *red lines*) and *lower* (*blue lines*) panels represent the chromatograms from the reactions with or without MM_0014, respectively. Extracted ions, *m/z* 273.4 and 287.4 (in ranges of ±0.5), correspond well with the [M+Na]^+^ of farnesyl ethyl ether and farnesyl propyl ether, respectively.

Instead of glycerol, small alcohols such as methanol, ethanol, 1-propanol, and 2-propanol were also tested for use as the acceptor substrate for MM_0014 reactions to elucidate the versatility of the enzyme. LC-ESI-MS analyses of the products from the reaction with FPP and 10% (v/v) ethanol, 1-propanol, or 2-propanol gave extracted ion peaks with *m/z* values corresponding to those of farnesyl ethyl ether, farnesyl 1-propyl ether, and farnesyl 2-propyl ether, respectively, suggesting the occurrence of *O*-prenylation reaction toward the alcohols (Fig. 7). 2-Propanol was likely the most reactive among the tested substrates as judged from the ion intensities of the presumed ether products, given that their ionization efficiencies were similar. Methanol seemed not a good acceptor substrate because the formation of farnesyl methyl ether was undetectable.

## Discussion

In the present study, we solved the substrate-free and substrate-bound crystal structures of a cPT from *M. mazei*, MM_0014. The co-FG+DMAPP structure revealed the binding mode of DMAPP as an acceptor substrate for irregular head-to-middle condensation. The observed conformations of the donor and acceptor substrates were basically similar to those in cPT homologs that specifically catalyze a head-to-middle condensation, such as lavandylyl pyrophosphate synthase, cyclolavandulyl pyrophosphate synthase, and isosesquilavandulyl pyrophosphate synthase (10, 11, 12). However, the 4.3 Å distance between the C1 of DMAPP at the donor site and the C2 of DMAPP at the acceptor site in MM_0014 was significantly longer than the distances between the corresponding atoms in cyclolavandulyl pyrophosphate synthase (3.7 Å in 5YGK) and isosesquilavandulyl pyrophosphate synthase (3.9 Å in 5YGK) (Fig. S4). This difference in distance could be why MM_0014 cannot catalyze the head-to-middle condensation between two DMAPP molecules, while it can catalyze the head-to-tail condensation between DMAPP and IPP (13). Although the IPP-binding structure of MM_0014 (free+IPP) did not provide verifiable information about distance, we inferred the cause for the substrate specificity of MM_0014 toward IPP based on the DMAPP-binding structure, as described above.

As far as we could ascertain, no cPT-family enzyme is known to catalyze *O*-prenylation. Even when including the hydrolysis of prenyl pyrophosphate, which can be regarded as the *O*-prenylation toward water, there are only two examples: tuberculosinol synthase from *Mycobacterium tuberculosis* and kolavelool synthase from *Herpetosiphon aurantiacus*, which are terpene synthases specifically catalyzing the hydrolysis of the allylic pyrophosphate substrates (19, 20). Thus, it was surprising that MM_0014, which originally catalyzed *C*-prenylation on the alkenyl carbon of IPP, could accept simple molecules such as glycerol, ethanol, 1-propanol, 2-propanol, and also H_2_O as acceptor substrates for *O*-prenylation. Catalysis of both *C*- and *O*-prenylation of the same or similar substrates has been reported with some aromatic prenyltransferases (21, 22, 23, 24, 25), but it is uncommon in other prenyltransferases such as cPT. The *O*-prenylation of alcohol is, however, an unlikely physiological reaction for MM_0014. It should be mentioned that *O*-prenylation of the glycerol moiety in archaeal membrane lipids is known to be catalyzed by other types of prenyltransferases (26) and that *M. mazei* possesses the putative genes of the enzymes.

**Figure 8:**
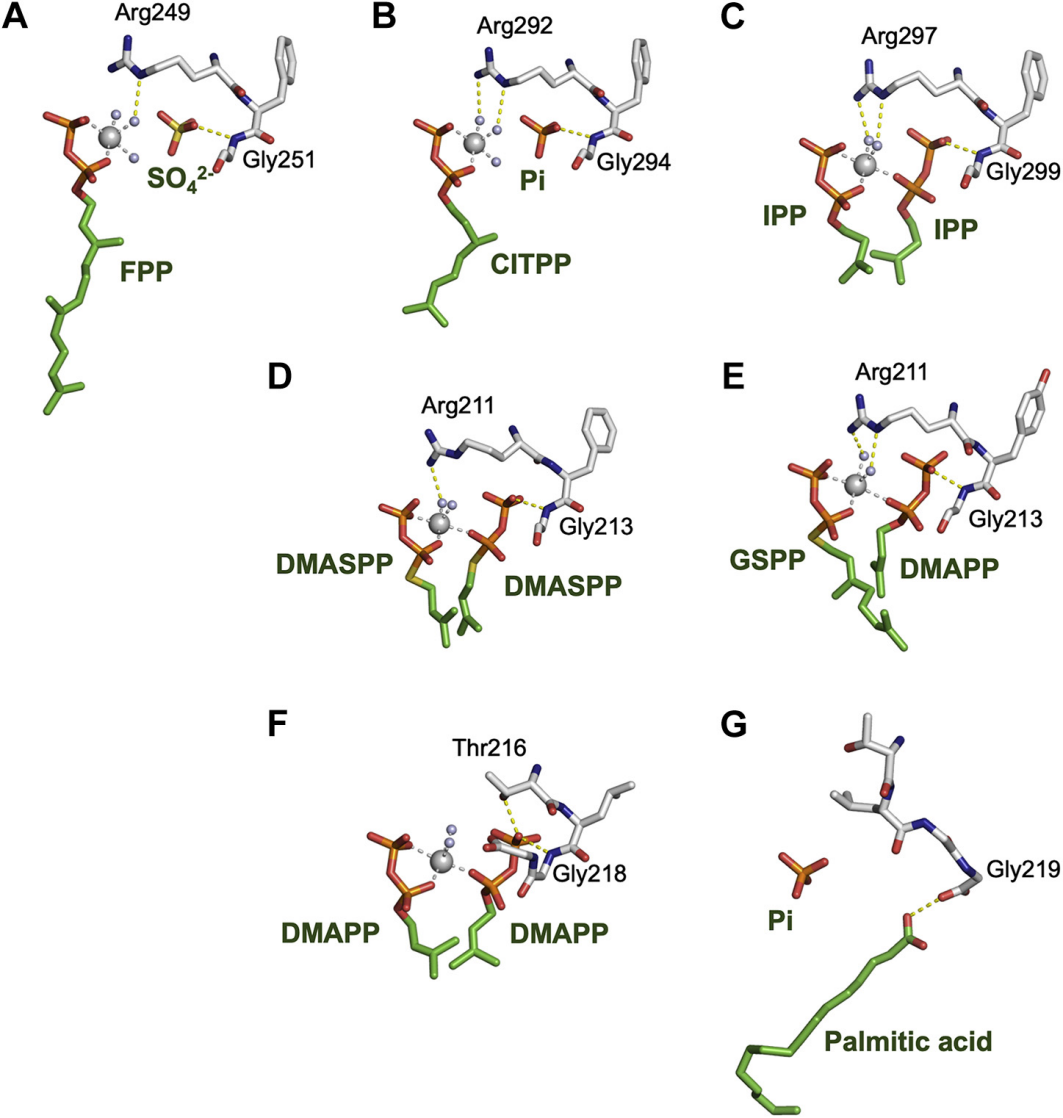
**Interactions between ligands and residues in C-terminal motif.** Residues in the RXG motif of cPTs (*A*–*E*) and the TLGG sequence of MM_0014 (*F* and *G*) and ligands/substrates are shown as *stick models*. Mg^2+^ ions and coordinated-water molecules are shown as *large* and *small spheres*, respectively. Coordinate and hydrogen bonds are shown as *gray* and *yellow dotted lines*, respectively. *A*, *Staphylococcus aureus* undecaprenyl pyrophosphate synthase in complex with FPP and sulfate ion (PDB code: 4H8E). *B*, *Mycobacterium tuberculosis* decaprenyl pyrophosphate synthase in complex with citronellyl pyrophosphate (CITPP) and phosphate ion (PDB code: 2VG3). *C*, *Solanum habrochaites* (*Z,Z*)-farnesyl pyrophosphate synthase in complex with IPP and Mg^2+^ ion (PDB code: 5HXP). *D*, *Streptomyces* sp. CL190 cyclolavandulyl pyrophosphate synthase in complex with dimethylallyl S-thiolopyrophosphate (DMASPP) and Mg^2+^ ion (PDB code: 5YGK). *E*, *Streptomyces* sp. CNH189 isosesquilavandulyl pyrophosphate synthase in complex with geranyl S-thiolopyrophosphate (GSPP), DMAPP and Mg^2+^ ion (PDB code: 5XK9). *F*, *M. mazei* MM_0014 in complex with FG and DMAPP (co-FG+DMAPP). *G*, *M. mazei* substrate-free MM_0014 subunit B. A palmitic acid binding in the cavity is shown.

Considering the high concentration of alcohols used in the reactions of MM_0014, the observed *O*-prenylation reactions could be regarded as insufficient protection of the catalytic site against solvents. A previous study that utilized a fluoro-substituted FPP analog suggested that the prenyltransfer reaction catalyzed by cPT proceeds *via* an S_N_2 mechanism (16). If it is applicable to MM_0014, a solvent molecule must be at the acceptor-binding site when the reaction occurs, and the nucleophilic attack from the hydroxyl group of the molecule must occur simultaneously with the elimination of the pyrophosphate group from the donor prenyl pyrophosphate. Given that a closed conformation is needed to fix the substrates and initiate the prenyltransfer reaction, MM_0014 could have the ability to present a closed conformation even when it holds alcohol instead of a proper acceptor substrate such as IPP. It is noteworthy that MM_0014 has a distinct feature in the C-terminal region, which plays the main role in the closure of the cavity. MM_0014 and its close relatives lack the RXG motif, which is highly conserved in the C-termini of homodimer-type cPT family enzymes (27) (Fig. S5). In the crystal structures of several cPT family enzymes with C-termini that are visible, such as *Staphylococcus aureus* undecaprenyl pyrophosphate synthase (4H8E), *M. tuberculosis* decaprenyl pyrophosphate synthase (2VG3), *S. habrochaites* (*Z,Z*)-farnesyl pyrophosphate synthase (5HXP), *Streptomyces* sp. CL190 cyclolavandulyl pyrophosphate synthase (5YGK), and *Streptomyces* sp. CNH189 isosesquilavandulyl pyrophosphate synthase (5XK9), the arginine residue in the RXG motif coordinates with Mg^2+^ ion *via* water, while the main chain nitrogen of the glycine residue interacts with the β-phosphate of the acceptor substrate in some of the structures (Fig. 8, *A*–*E*). The lack of the arginine residue could be why MM_0014 can accept solvent molecules that have no pyrophosphate group. The interaction between the C-terminal sequence of MM_0014, TLGG, and the substrates of prenyl condensation reactions seems weak; only the main chain nitrogen of Gly223 and the hydroxyl group of Thr221 likely coordinate with the acceptor substrate DMAPP in the DMAPP-binding structure (Fig. 8*F*). In the substrate-free structure, however, the carboxyl group of the C-terminal Gly219 residue interacts with the carboxyl group of the fatty acid ligand at the acceptor-binding site (Fig. 8*G*). When the two structures are superposed, the position of the Gly219 carboxyl group in the substrate-free structure overlaps the β-phosphate of DMAPP at the accepter-binding site in the DMAPP-binding structure, while the fatty acid carboxyl group overlaps the α-phosphate. This arrangement implies the possibility that the C-terminal carboxyl group might coordinate with Mg^2+^ ion, along with the pyrophosphate group of prenyl pyrophosphate at the donor-binding site, in the absence of both a fatty acid ligand and an acceptor substrate with a pyrophosphate group. With this characteristic C-terminal sequence, MM_0014 could form a closed conformation even when alcohol exists at the acceptor-binding site.

The physiological role of MM_0014 remains unclear. Regular prenyl condensation reactions catalyzed by the enzyme from prenyl pyrophosphates and IPP yield C_25–40_ polyprenyl pyrophosphates that are shorter than the C_50_ polyprenyl chain of the putative glycosyl carrier lipid extracted from *M. mazei* cells (13). Because the other cPT paralogs from *M. mazei*, MM_0618 and MM_1083, are known to form heteromeric cPT and are used to synthesize C_45–50_ products, they are likely responsible for glycosyl carrier lipid biosynthesis (13). It is possible, however, that the shorter polyprenyl pyrophosphates produced by MM_0014 are also utilized as the precursors of glycosyl carrier lipids as in the case of *Saccharomyces cerevisiae*, in which two cPTs give products with different chain lengths that are used as needed to adapt to the growth phase of the cells (28, 29). Nevertheless, the “loose” substrate specificity of MM_0014 accepts not only the isomer of IPP, *i.e.*, DMAPP, but also small alcohols, as supported by the data from this study. This suggests that the enzyme could play a different role and produce an unidentified isoprenoid compound in the cells of *M. mazei*.

## Experimental procedures

### Materials

Precoated reversed-phase TLC plates, RP-18 F_254S_, were purchased from Merck Millipore. [1-^14^C]IPP was purchased from American Radiolabeled Chemicals, Inc. Nonlabeled IPP, DMAPP, and FPP were donated by Dr Chikara Ohto, Toyota Motor Co. GPP and GGPP were purchased from Sigma-Aldrich.

### Expression and purification of recombinant cPT homologs from *M. mazei*

The recombinant expression of MM_0014 fused with an N-terminal thioredoxin-polyhistidine-tag in *E. coli* and its partial purification by affinity-chromatography were performed as described elsewhere (13). *E. coli* C41(DE3) strain transformed with the plasmid pET48b-MM_0014 was cultivated in LB medium supplemented with 50 mg/l kanamycin at 37 °C until log phase, followed by overnight cultivation at 22 °C after addition of 0.5 mM IPTG. The cells were harvested and then disrupted by sonication. Polyhistidine-tagged MM_0014 was purified from the cell-free extract using 1 ml HisTrap FF crude column (GE healthcare). The buffer exchange of the affinity-purified MM_0014 solution into buffer A (20 mM sodium phosphate buffer, pH7.4, containing 0.5 M NaCl) was performed with a Vivaspin Turbo 15 (10,000 MWCO) ultracentrifugation unit (Sartorius). After the solution was concentrated, ten units of HRV-3C protease were added per 1 mg of tagged MM_0014. After incubation at 4 °C for 24 h, the solution was loaded onto a HisTrap FF 1 ml Ni-NTA affinity column equilibrated with buffer A, and the flow-through fraction containing tag-free MM_0014 was recovered. The tag-free MM_0014 was used for enzymatic characterization such as product analysis. The cleavage of the tag was confirmed *via* 12% SDS-PAGE (Fig. S6). The buffer of the flow-through fraction was exchanged with buffer B (10 mM Tris-HCl, pH8.0, containing 150 mM NaCl) and concentrated using a Vivaspin Turbo 4 (10,000 MWCO) ultracentrifugation unit (Sartorius), and the concentrated solution of MM_0014 was loaded onto a HiLoad 16/600 Superdex 200 prepgrade gel-filtration column (GE Healthcare) and eluted with buffer B at a flow rate of 1 ml/min. The elution of protein was monitored by UV absorption at 280 nm, and the fractions at 75 to 90 min were recovered. The purity of the protein was confirmed *via* 12% SDS-PAGE (Fig. S6). After being concentrated to 17 to 19 mg/ml with a Vivaspin Turbo 4 (10,000 MWCO) ultracentrifuge unit, purified MM_0014 was utilized for crystallization.

### Crystallization, X-ray data collection, and refinement

The purified MM_0014 was crystallized *via* a hanging drop vapor diffusion method. Crystals of substrate-free MM_0014 (the crystal-type “substrate-free” in Table 1) were obtained using a 3:1 mixture of Index No. 64 reagent [0.005 M cobalt (II) chloride hexahydrate, 0.0005 M nickel (II) chloride hexahydrate, 0.005 M cadmium chloride hydrate, 0.005 M magnesium chloride hexahydrate, 0.1 M HEPES, pH7.5, 12% polyethylene glycol 3350, Hampton Research] and glycerol as a reservoir solution. A drop was formed by mixing 2 μl of purified MM_0014 and 3 μl of the reservoir solution, and crystals were grown at 25 °C. Crystals of MM_0014 complexed with FG (co-FG) were obtained using a 3:1 mixture of Index No. 89 reagent (0.1 M succinic acid, pH7.0, 15% w/v polyethylene glycol 3350, Hampton Research) and glycerol as a reservoir solution. A drop was formed by mixing 2 μl of purified MM_0014, 0.25 μl of 20 mM FPP, 0.25 μl of 10 mM MgCl_2_, and 4 μl of the reservoir solution, and crystals were grown at 20 °C.

**Table 1:** Data collection and refinement statistics

Crystal type	Substrate-free	Free+IPP	Free+PPi	Co-FG	Co-FG+DMAPP
Data collection and processing statistics					
Beam line	NW-12A	NW-12A	NW-12A	BL-5A	BL-5A
Space group	*C*222_1_	*C*222_1_	*C*222_1_	*P*2_1_2_1_2_1_	*P*2_1_2_1_2_1_
Unit cell dimension (Å)					
*a* (Å)	80.02	79.94	79.78	99.05	98.85
*b* (Å)	100.44	100.42	100.22	99.22	98.91
*c* (Å)	130.52	129.56	130.09	193.88	193.85
Wavelength (Å)	1.000	1.000	1.000	1.000	1.000
Resolution (Å)^a^	65.3–1.69 (1.78–1.69)	64.8–1.98 (2.09–1.98)	65.0–2.28 (2.40–2.28)	48.5–1.72 (1.75–1.72)	48.46–1.91 (1.94–1.91)
Total reflections	781,543	509,434	336,815	4,519,733	3,287,088
Unique reflections	59,466	36,516	24,258	202,517	147,512
*I*/σ*I*^a^	20.1 (3.0)	22.1 (3.9)	19.3 (3.7)	42.6 (2.1)	33.6 (2.2)
Redundancy^a^	13.1 (12.5)	14.0 (13.2)	13.9 (13.2)	22.3 (21.7)	22.3 (22.6)
Completeness^a^ (%)	99.7 (99.8)	99.8 (99.8)	100 (100)	100 (100)	100 (99.7)
*R*_merge_^a^^,^^b^ (%)	7.2 (81.6)	7.1 (66.0)	9.5 (69.7)	5.2 (184)	6.6 (205)
CC_1/2_^a^	0.999 (0.846)	0.999 (0.856)	0.999 (0.858)	1.000 (0.672)	1.000 (0.688)
Refinement statistics					
Resolution	65.3–1.69	39.7–1.98	65.0–2.28	48.1–1.72	47.9–1.91
Protein atoms	3384	3356	3406	13,865	13,896
Protein molecules	2	2	2	8	8
Ligand atoms	28	42	33	376	272
Ligand molecules	3	4	4	24	28
Water molecule	298	147	117	781	485
*R*_work_/*R*_free_ (%)	20.9/24.8	22.0/25.9	22.0/25.8	19.7/21.3	19.6/21.9
Root mean square deviations					
Bond lengths (Å)	0.011	0.009	0.003	0.004	0.005
Bond angles (°)	1.666	1.504	1.010	1.271	1.305
Ramachandran statistics (%)					
Residues in favored region	97.5	98.0	97.8	98.3	98.4
Residues in allowed region	2.5	1.7	1.9	1.6	1.5
Residues in outlier region	0	0.2	0.2	0.1	0.1

a Numbers in parentheses are for the highest shell.

b *R*_merge_ = 100Σ|*I* - <*I*>|/Σ *I*, where *I* is the observed intensity and <*I*> is the average intensity from multiple observations of symmetry-related reflections.

Crystals that yielded the free+IPP and free+PPi structures (called crystal-types “free+IPP” and “free+PPi”, respectively) were obtained by soaking the substrate-free crystals in the crystallization drops containing 10 mM IPP for 12 h, or 10 mM DMAPP for 10 min, respectively (Table 1). A crystal-type “co-FG+DMAPP,” which yielded the co-FG+DMAPP structure, was obtained by soaking the co-FG crystals in the crystallization drops containing 10 mM DMAPP for 39 min. Each of the crystals was then frozen with liquid nitrogen. X-ray diffraction data from substrate-free, free+IPP, and free+PPi crystals were collected using synchrotron radiation on a beamline AR-NW12 A at the Photon Factory using Quantum Q270 (Area Detector Systems Corporation) detector. X-ray diffraction data from co-FG and co-FG+DMAPP crystals were collected using synchrotron radiation on a beamline BL-5A at the Photon Factory using PILATUS3 S6M (Dectris) detector. All measurements were performed at 100K with λ = 1.000 Å. The datasets from the co-FG and co-FG+DMAPP crystals were processed *via* XDS (30) and scaled with Aimless (31), while that from the others were processed with Mosflm (32) and SCALA (33). These datasets were categorized as two types of space groups, *C*222_1_ and *P*2_1_2_1_2_1_, with two and eight protein molecules per asymmetric unit, respectively. Datasets from the substrate-free, free+IPP, and free+PPi types of crystals belong to the space group *C*222_1_, while those from co-FG and co-FG+DMAPP belong to the space groups *P*2_1_2_1_2_1_. Phase information for the substrate-free structure was calculated *via* molecular replacement using the MrBUMP program (34). The structure was then modeled by coot (35) and refined using Refmac (36) with 5% of the data was set aside as a free data set. The other structures were solved *via* the molecular replacement method using the substrate-free structure. The molecular replacements were performed using the Molrep CCP4 suite (37) and refined with Refmac (36). Models of the ligands were fitted into the ligand-binding sites according to difference electron density maps (Fig. 3*D* and Fig. S2). The refinement statistics are listed in Table 1. Figure 1, Figure 2, Figure 3, Figure 4, 7, and Figs. S1–S4 were produced using PyMOL (version 2.3) software (http://www.pymol.org).

### Radio-TLC analysis of *O*-prenyltransfer reaction products

The reaction mixture for the *O*-prenyltransfer reaction toward glycerol contained, in a final volume of 100 μl, 50 pmol [^14^C]FPP (110 Ci/mol), 10 μmol MOPS-NaOH, pH7.0, 0.5 μmol MgCl_2_, 100 pmol tag-free MM_0014, and 25% (v/v) glycerol. The enzymatic synthesis of [^14^C]FPP was performed as described elsewhere (38). The mixture was incubated for 1 h at 37 °C, and then hydrophobic compounds were extracted with 600 μl of *n*-pentane after the addition of 200 μl of water. The pentane layer was recovered and dried under a N_2_ stream. The dried residue was dissolved with *n*-pentane and spotted on a RP-18 F_254S_ reversed-phase TLC plate (Merck Millipore) to be developed with acetone/H_2_O (9:1). The distribution of radioactivity on the plate was visualized with a Typhoon FLA9000 multifunctional scanner (GE Healthcare).

### LC-MS analysis of *O*-prenyltransfer reaction products

The reaction mixture for the *O*-prenyltransfer reaction toward alcohols contained, in a final volume of 200 μl in one tube, 50 nmol FPP, 20 μmol 3-morpholinopropanesulfonic acid (MOPS)-NaOH, pH7.0, 1 μmol MgCl_2_, 500 pmol tag-free MM_0014, and an acceptor substrate [25% (v/v) glycerol or 10% (v/v) small alcohol (methanol, ethanol, 1-propanol, or 2-propanol)]. After incubation for 4 h at 37 °C, the reaction mixtures from six tubes were gathered. The reaction products were extracted with 1.5 ml of *n*-pentane and dried under a N_2_ stream. Dried residue was dissolved with 100 μl of a 9:1 (v/v) mixture of methanol and 10 mg/l CH_3_COONa.

LC-ESI-MS analysis was performed in the positive-ion mode with an Esquire 3000 ion trap system (Bruker Daltonics) connected to an Agilent 1100 Series HPLC (Agilent Technologies). The compounds eluted from an InertSustain C18 (5 μm, 2.1 × 150 mm, GL Sciences) were detected *via* UV absorption at 210 nm and ESI-MS in the positive-ion mode. The mobile phase used for the analysis was made up of a mixture of solution A [the 9:1 (v/v) mixture of methanol and 10 mg/l CH_3_COONa] and solution B (isopropanol) at a flow rate of 0.2 ml·min^−1^. Sample elution was performed with a 0% ratio of B for the initial 15 min and then 90% for the next 20 min. After each analysis, the column was equilibrated with 0% B. Standard MS parameters were used: sheath gas, N_2_ of 30 psi; dry gas, N_2_ of 7.0 l·min^−1^, 350 °C; scanning range, 50 to 1000 m/z; scan speed, 13,000 m/z·s^−1^; ion charge control target, 50,000; maximum accumulation time, 200 ms; averages, 10; and, rolling averaging, 1.

## Data availability

The structures presented in this paper have all been deposited in the Protein Data Bank with the following codes: 7CAQ, 7CAR, 7CAS, 7CC3, and 7CAV. All remaining data are contained within the article.

## Supporting information

This article contains supporting information.

## Conflict of interest

The authors declare no conflicts of interest in regard to this article.
