# Supplementary material for: A versatile cis-prenyltransferase from Methanosarcina mazei catalyzes both C- and O-prenylations
Source: J Biol Chem. 2021 Apr 17;296:100679. doi: 10.1016/j.jbc.2021.100679 (PMC8131916; doi:10.1016/j.jbc.2021.100679)
Supplement: Supplemental Figures S1–S6 [file mmc1.pdf]

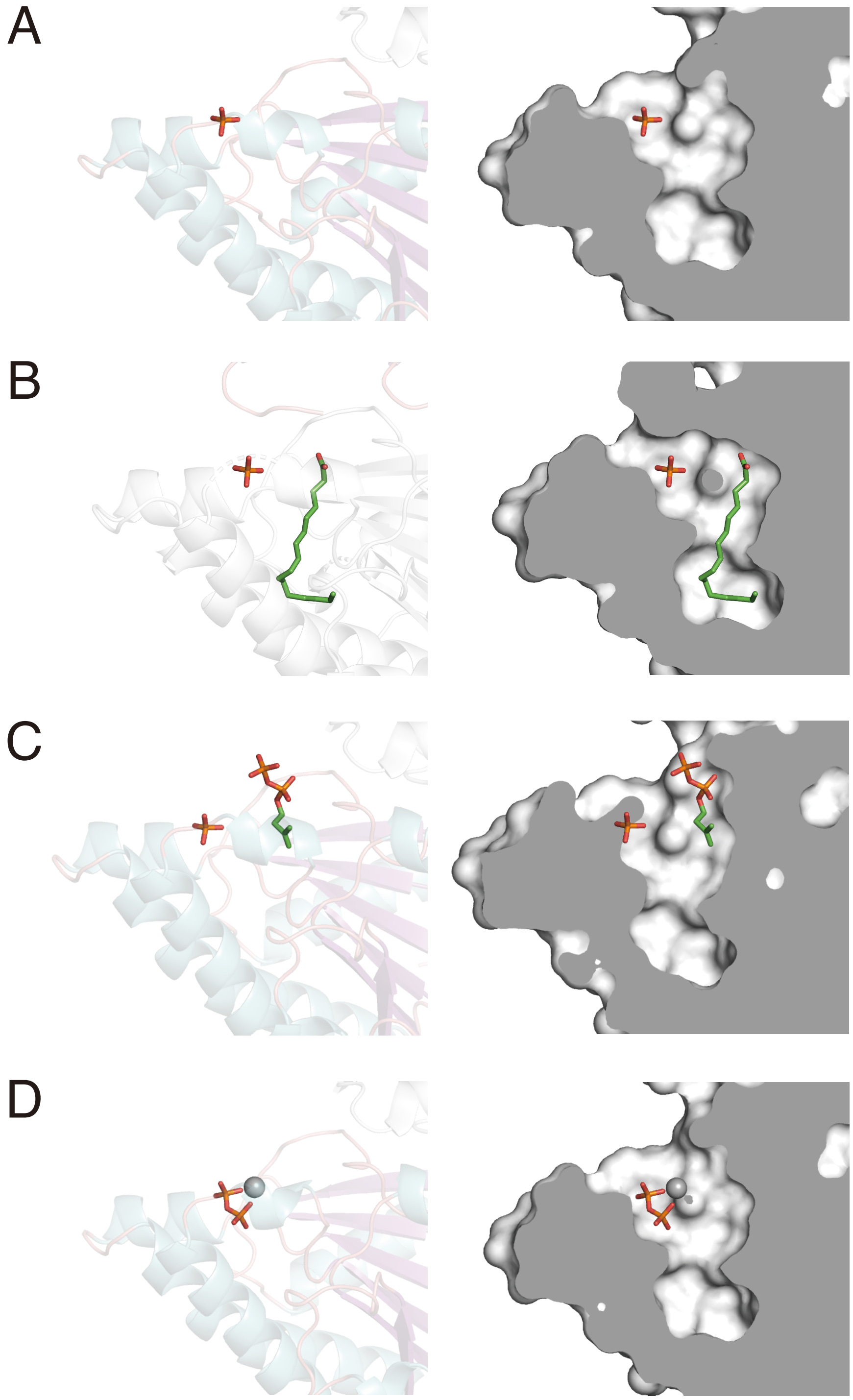
A
B
C
D

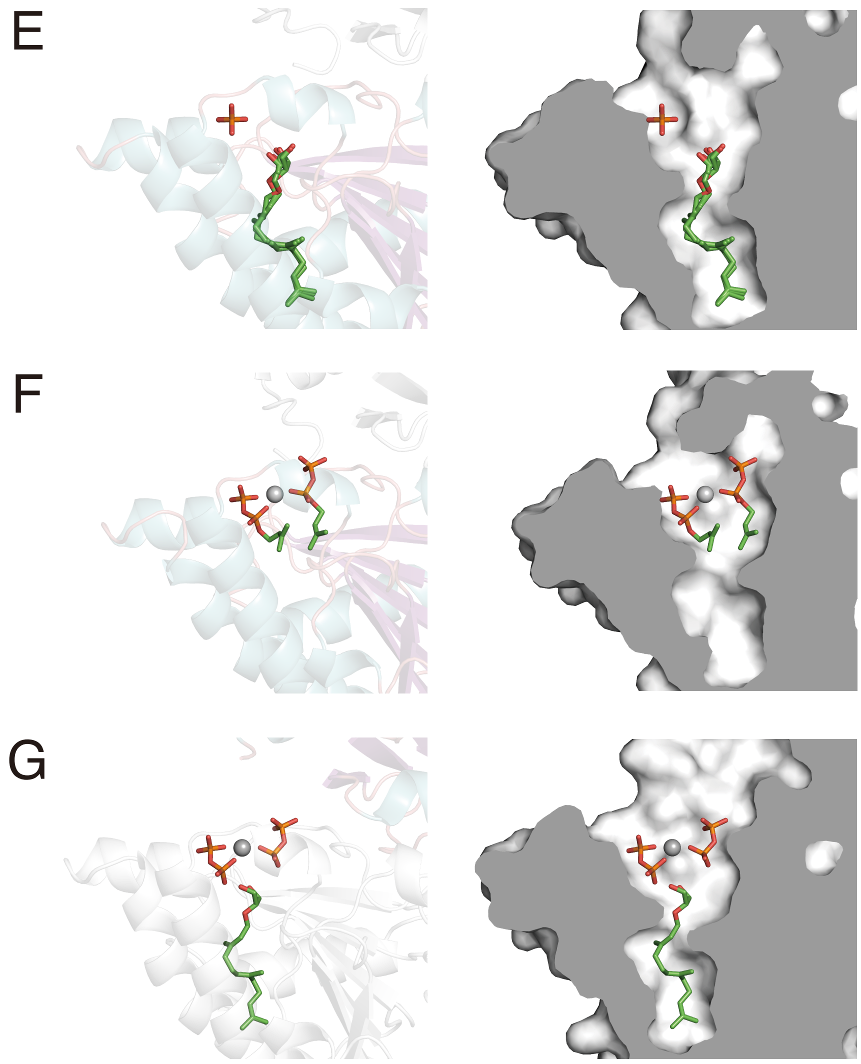

Supplementary Figure S1. Comparison of ligand-binding cavities in complex structures of MM_0014. Left and right panels show ribbon model and surface representations of the cavity structures, respectively. Subunits A and B in ribbon models are colored cyan and light-gray, respectively. Ligand molecules and Mg^{2+} ion are shown as stick models and a sphere, respectively. (A) Subunit A of the substrate-free structure. (B) Subunit B of the substrate-free structure. (C) Subunit A of the IPP-binding structure (free+IPP). (D) Subunit A of the pyrophosphate-binding structure (free+PPi). (E) The FG-binding structure (co-FG). (F, G) DMAPP-binding subunit (F) and pyrophosphate/FG-binding subunit (G) observed in the co-FG+DMAPP structure. Panels A-D correspond with Fig. 2A-D, respectively. Panel E corresponds with Fig. 3. Panels F and G correspond with upper and lower panels in Fig. 4A, respectively.

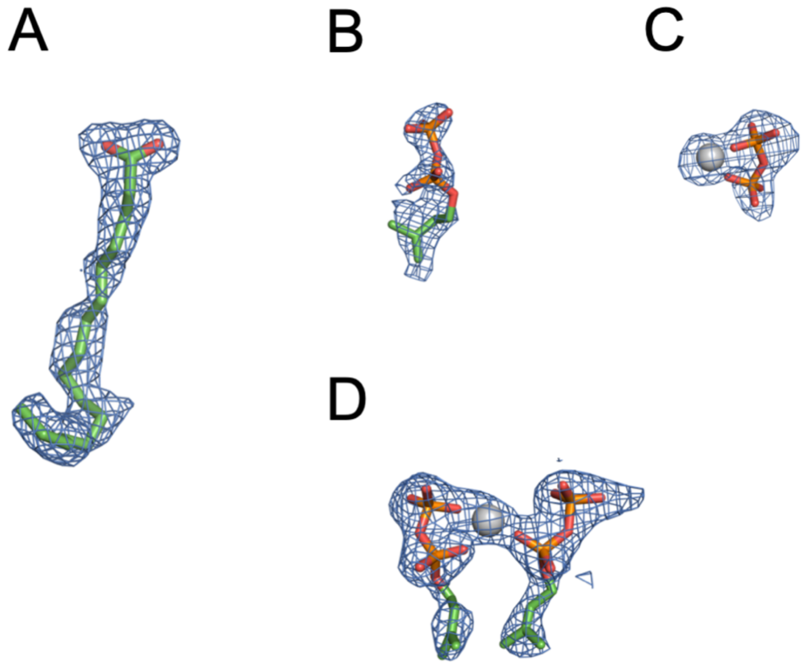

Supplementary Figure S2. F_o-F_c omit electron map (blue) for ligands. Ligand molecules and Mg^{2+} ions are shown as stick models and spheres, respectively. Palmitic acid (A), IPP (B), PPi and Mg^{2+} (C), and DMAPP and Mg^{2+} (D) from the crystal types of substrate-free, free+IPP, free+PPi, and co-FG+DMAPP are shown, respectively. The contour levels of the omit maps in the panels from A to D are 3.0σ, 2.5σ, 3.0σ, and 2.5σ, respectively.

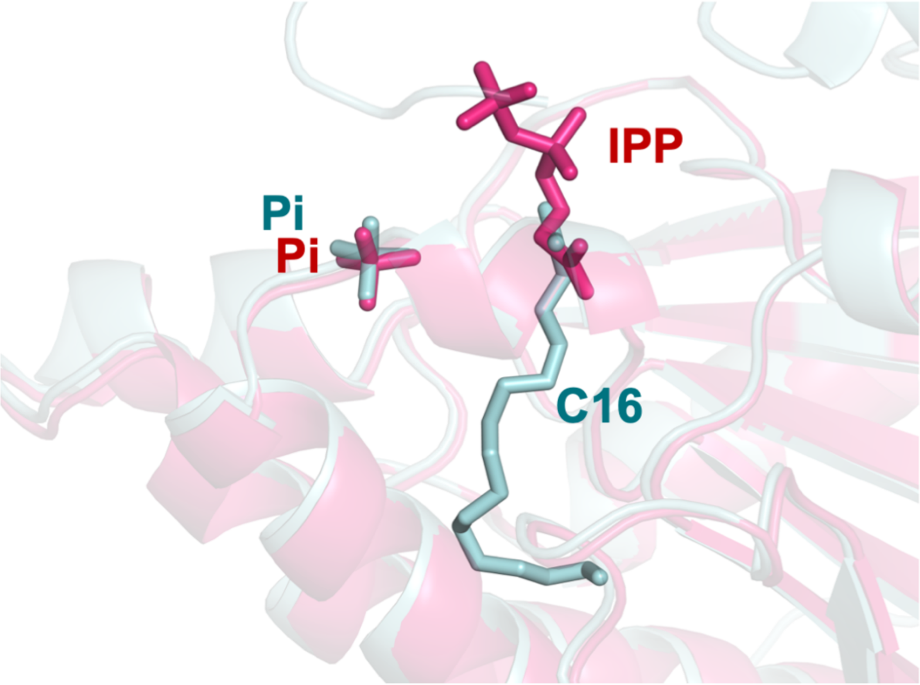

Supplementary Figure S3. Superposition of Subunit A and B of IPP-binding structure (free+IPP). Subunit A and B are colored pink and cyan, respectively. IPP, palmitic acid (C16), and binding-phosphates (Pi) are shown as stick models.

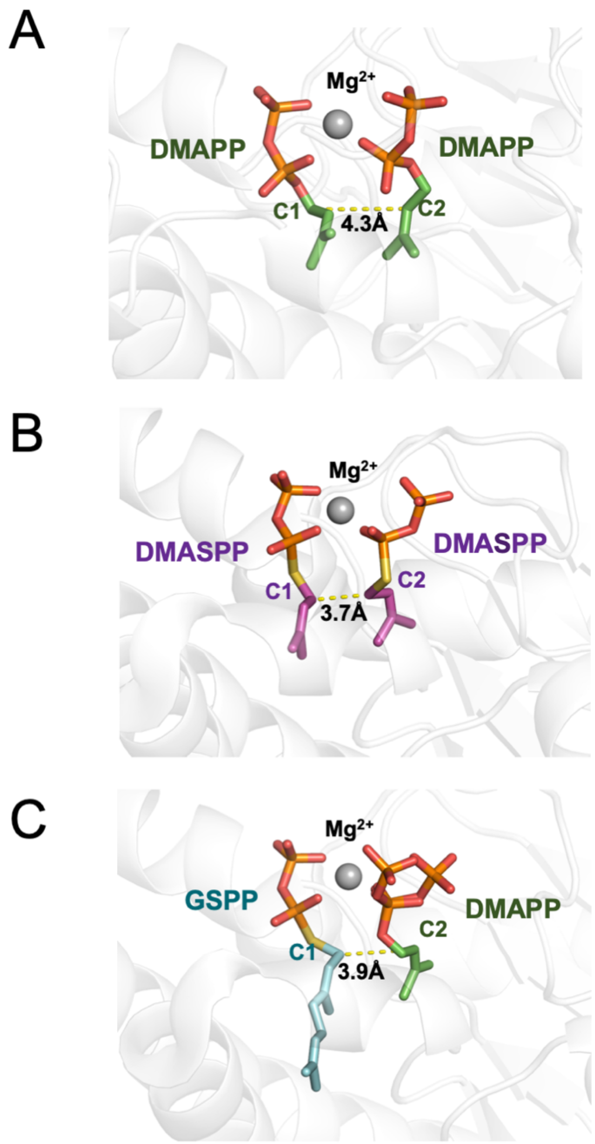

Supplementary Figure S4. Comparison with ligand-binding structures of the cPTs. (A) Versatile cPT MM_0014 from *M. mazei* in complex with DMAPP (co-FG+DMAPP). (B) Cyclolavandulyl pyrophosphate synthase from *Streptomyces* sp. CL190 in complex with DMASPP (PDB code: 5YGK). (C) Isosesquilavandulyl pyrophosphate synthase from *Streptomyces* sp. strain CNH-189 in complex with GSPP and DMAPP (PDB code: 5XK9). Ligand molecules and Mg^{2+} ions are shown as stick models and spheres, respectively. Protein structures are colored semi-translucent light-gray.

```
SaUPS      239 ACHAFAARERKFGGLPTPPANSQARRPSAPKATR 272
EcoUPS     232 ALNAFANRERRFGG--TEPGDE----------TA 253
MluUPS     235 CISIYQNRHRRFGGL------------------- 249
SauUPS     239 CIKIYQSRQRRFGGLSEE---------------- 256
MtuDPS     282 ACEEYASRTRRFG-------------------SA 296
ShazzFPS   287 AIINFQQRHRRFGG-----------------HTY 303
MtuzeFPS   249 ALRDYSARHRSYG--------------------R 262
MM_0014    206 ALEFYQNQDITLGG-------------------- 219
MA1831     211 ALEFYQDQDITLGG-------------------- 224
ApeUPS     206 AVQWFSTRRRPMG--------------------R 219
StrCLPS    201 ALKDLRARDRRFGGYPA----------------- 217
StrISLPS   216 ALEDLRERDRRYGLYPV----------------- 232
                           ___
                           RXG motif
```

Supplementary Figure S5. Alignment of the C-terminal sequences of homodimer-type cPT family enzymes. Arginine and lysine residues are shown in blue, and glycine residues are shown in red. SaUPS, *Sulfolobus acidocaldarius* undecaprenyl pyrophosphate synthase; EcoUPS, *E. coli* undecaprenyl pyrophosphate synthase; MluUPS, *Micrococcus luteus* undecaprenyl pyrophosphate synthase; SauUPS, *Staphylococcus aureus* undecaprenyl pyrophosphate synthase; ShazzFPS, *Solanum habrochaites* (*Z,Z*)-FPS; MtuzeFPS, *Mycobacterium tuberculosis* (*Z,E*)-FPS; ApeUPS, *Aeropyrum pernix* undecaprenyl pyrophosphate synthase; StrCLPS, *Streptomyces* sp. CL190 cyclolavandulyl pyrophosphate synthase; StrISLPS, *Streptomyces* sp. CNH189 isosesquilavandulyl pyrophosphate synthase. Sequence alignment was performed with CLC Sequence Viewer 7 (CLC bio, Denmark).

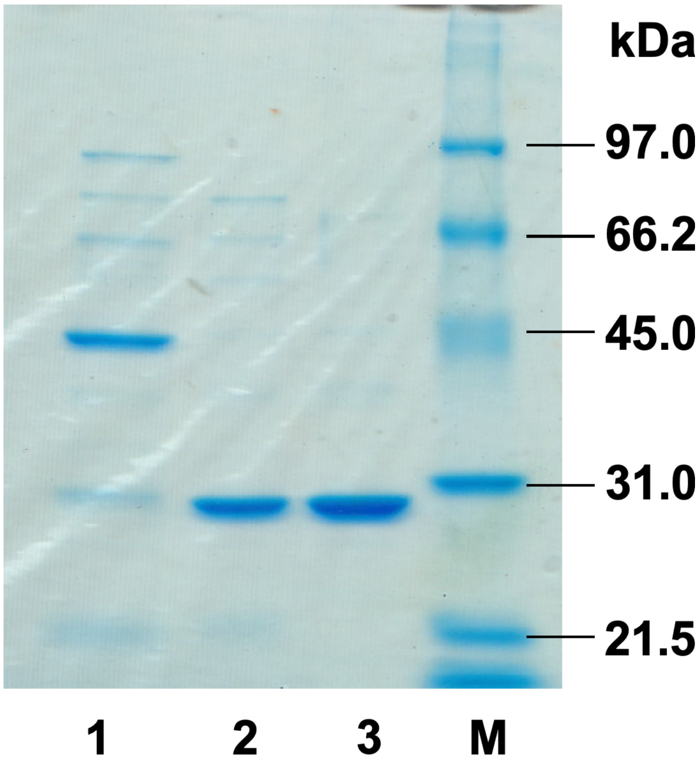

Supplementary Figure S6. SDS-PAGE analysis of purified MM_0014. Lane 1, Affinity-purified polyhistidine-tagged MM_0014; lane 2, tag-free MM_0014 recovered from the flow-through fraction of the second affinity chromatography; lane 3, purified MM_0014 after gel-filtration chromatography; lane M, protein molecular weight marker.
